# Synergistic Autophagy-Related Mechanisms of Protection Against Brain Aging and AD: Cellular Pathways and Therapeutic Strategies

**DOI:** 10.3390/ph18060829

**Published:** 2025-06-01

**Authors:** Bogdan Cordos, Amelia Tero-Vescan, Ian N. Hampson, Anthony W. Oliver, Mark Slevin

**Affiliations:** 1 Center of Experimental and Imaging Studies, George Emil Palade University of Medicine, Pharmacy, Science and Technology of Târgu Mureș, 38th Gh. Marinescu Street, 540139 Târgu Mureş, Romania; bogdan.cordos@umfst.ro; 2 Doctoral School of Medicine and Pharmacy, George Emil Palade University of Medicine, Pharmacy, Science and Technology of Targu Mures, 540139 Târgu Mureş, Romania; 3 Department of Biochemistry, George Emil Palade University of Medicine, Pharmacy, Science and Technology of Târgu Mureș, 38th Gh. Marinescu Street, 540139 Târgu Mureş, Romania; amelia.tero-vescan@umfst.ro; 4 Division of Cancer Sciences, University of Manchester, Oxford Rd, Manchester M13 9WL, UK; ian.hampson@manchester.ac.uk; 5 Ravan Bio Ltd., Unit 7A Kilburn House, Lloyd Street N, Manchester M15 6SE, UK; awo@ravanbio.com; 6 Center for Advanced Medical and Pharmaceutical Research, George Emil Palade University of Medicine, Pharmacy, Science and Technology of Târgu Mureș, 38th Gh. Marinescu Street, 540139 Târgu Mureş, Romania

**Keywords:** Alzheimer’s disease, autophagy, neurodegeneration, metformin, benzimidazole, acetylsalicylic acid

## Abstract

Brain aging is driven by interconnected processes, including impaired autophagy, chronic inflammation, mitochondrial dysfunction, and cellular senescence, all of which contribute to neurovascular decline and neurodegenerative diseases such as Alzheimer’s disease (AD). Targeting these mechanisms simultaneously offers a promising therapeutic approach. This review explores the rationale for combining metformin, benzimidazole derivatives, phosphodiesterase-5 (PDE5), and acetylsalicylic acid (ASA) as a multi-targeted strategy to restore proteostasis, reduce senescence-associated secretory phenotype (SASP) factors, and enhance mitochondrial and lysosomal function. Metformin activates AMP-activated protein kinase (AMPK) and promotes autophagy initiation and chaperone-mediated autophagy, whilst benzimidazole derivatives enhance lysosomal fusion through JIP4–TRPML1 pathways independently of mTOR signaling; and ASA augments autophagic flux while suppressing NF-κB-driven inflammation and promoting specialized pro-resolving mediator pathways. This combinatorial approach targets both upstream autophagy initiation and downstream autophagosome–lysosome fusion, while concurrently attenuating inflammation and cellular senescence. Patient stratification based on the biomarkers of autophagy impairment, inflammation, and metabolic dysfunction could optimize therapeutic responses. While this strategy shows strong preclinical promise, careful attention to timing, dosing, and cell-specific responses is crucial to maximize benefits and avoid adverse effects. Future studies integrating biomarker-guided precision medicine frameworks are essential to validate the potential of this therapeutic combination in preventing or slowing cognitive decline and promoting healthy brain aging.

## 1. Introduction

Aging is a complex biological process that affects multiple organ systems, with the brain being particularly vulnerable to its progressive effects. Brain aging is marked by a gradual decline in neuronal function, impaired synaptic plasticity, and reduced neu-rovascular integrity, all of which contribute to cognitive deterioration and an increased risk of neurodegenerative diseases such as Alzheimer’s disease (AD), Parkinson’s disease (PD), and vascular dementia [1]. While some degree of cognitive slowing is expected with age, the accumulation of cellular and molecular damage can exacerbate neuronal dysfunction and compromise brain homeostasis. These age-related changes manifest in memory loss, decreased processing speed, and impairments in executive function, making brain aging a significant challenge in an increasingly aging global population.

At the cellular and molecular levels, several key processes drive brain aging. Chronic neuroinflammation, driven by the persistent activation of microglia and astrocytes, leads to the excessive release of pro-inflammatory cytokines, exacerbating neuronal damage [2]. Additionally, cellular senescence, characterized by the accumulation of aged, non-dividing cells and pro-inflammatory cell phenotype expression, contributes to neurodegenerative pathology through transformation to the neuronal–glial senescence-associated secretory phenotype (SASP), the concomitant secretion of pro-inflammatory cytokines, and promoting oxidative stress. Mitochondrial dysfunction further accelerates aging by impairing ATP production and increasing reactive oxygen species (ROS), which damage cellular structures. Moreover, the decline in autophagy and proteostasis, which are essential for the removal of toxic protein aggregates, contributes critically to the accumulation of misfolded proteins such as β-amyloid (Aβ) and tau in neurodegenerative diseases. Understanding the interplay between these mechanisms is therefore critical for developing targeted interventions to mitigate brain aging and preserve cognitive function [3].

Several promising compounds, including metformin, rapamycin, senolytics, phosphodiesterase-5 (PDE5) inhibitors, benzimidazoles, and acetylsalicylic acid (ASA), have demonstrated potential to modulate the key cellular pathways involved in brain aging, particularly autophagy and senescence. These agents act through diverse but complementary mechanisms: enhancing autophagic flux, reducing senescent cell burden, improving mitochondrial function, and dampening neuroinflammation. Given the critical role of impaired proteostasis and chronic inflammation in the pathogenesis of AD and other neurodegenerative disorders, targeting these pathways offers a compelling strategy for neuroprotection. By elucidating the interconnected mechanisms underlying brain aging and presenting these compounds as potential modulators, this review highlights the rationale for combinatorial or synergistic therapies aimed at promoting cognitive resilience and delaying neurodegeneration.

## 2. Mismatch Between Senescence and Autophagy, a Critical Switch Initiating and Propagating Neurovascular Dysfunction and Blood–Brain Barrier Integrity

The blood–brain barrier (BBB) plays a fundamental role in maintaining cerebral homeostasis by regulating the selective transport of nutrients and preventing the entry of neurotoxic substances. Its integrity is crucial for protecting neural tissues from inflammatory insults, oxidative stress, and harmful circulating molecules. However, with aging and neurodegenerative diseases, the BBB becomes increasingly permeable, leading to cognitive decline and neuroinflammation. Endothelial dysfunction, pericyte degeneration, and increased cellular senescence contribute to this breakdown, disrupting neurovascular coupling and accelerating neuronal damage.

Among the cell types responsible for BBB function, pericytes play a critical role by stabilizing endothelial cells and regulating vascular permeability. Pericyte dysfunction and senescence result in vascular instability, leading to increased BBB permeability. Studies have shown that activating autophagy with rapamycin can reverse pericyte senescence and protect against BBB degradation [4]. Similarly, in AD models, senescent endothelial cells accumulate near the regions of BBB leakage, indicating that targeting vascular senescence with senolytics or BBB-stabilizing agents may be a potential strategy to slow disease progression [5].

Beyond endothelial and pericyte dysfunction, cellular senescence plays a pivotal role in BBB integrity loss through chronic inflammatory signaling. Senescent neurons exhibit increased oxidative stress, DNA damage, and epigenetic alterations, contributing to tau hyperphosphorylation and neuroinflammation in AD [6]. Astrocyte senescence, another key factor, disrupts neuronal metabolism and releases pro-inflammatory cytokines (e.g., Interleukin-6 (IL-6) and Tumor Necrosis Factor alpha (TNF-α)), further exacerbating neuronal dysfunction [7]. Likewise, senescent endothelial cells, central to BBB maintenance, lose their ability to regulate vascular integrity, leading to chronic neurovascular inflammation and cognitive impairment [5].

Microglia, the resident immune cells of the central nervous system, rely heavily on efficient autophagic machinery to maintain their homeostatic functions. Impaired autophagy in microglial cells results in chronic neuroinflammation characterized by excessive pro-inflammatory cytokine production, significantly reduced clearance of extracellular Aβ plaques, and subsequent synaptic dysfunction [8]. The published literature indicates that autophagic dysfunction promotes microglial polarization toward a pro-inflammatory M1 phenotype while suppressing the neuroprotective M2 phenotype, thereby creating a neurotoxic microenvironment conducive to neurodegeneration. Dysfunctional autophagy in microglia also compromises their phagocytic capacity, further impeding the clearance of neurotoxic protein aggregates from the brain parenchyma.

In AD, defective lysosomal function constitutes a critical component of the pathogenic cascade. Lysosomal dysfunction results in the aberrant accumulation of Aβ and hyperphosphorylated tau aggregates within neurons and the extracellular space, significantly accelerating neuronal degeneration and cognitive decline [9]. Therefore, the progressive extension of autophagic–lysosomal pathways creates a self-perpetuating cycle where protein aggregation further compromises cellular clearance mechanisms, ultimately leading to neuronal death.

Given the interconnected roles of cellular senescence and BBB dysfunction in aging and neurodegeneration, targeting senescence through autophagy (re)activation represents a promising therapeutic approach. Autophagy is essential for removing damaged organelles and protein aggregates, preventing the accumulation of senescent cells and the development of SASP-mediated inflammation. Enhancing autophagy through pharmacological agents or lifestyle interventions could help mitigate BBB permeability, preserve neurovascular integrity, and slow the progression of neurodegenerative disorders.

## 3. Mechanisms of Impaired Autophagy, Associated Neurodegeneration, and Novel Therapeutic Strategies for Protection of the Neurovascular Unit (NVU) and BBB

The pharmacological enhancement of autophagy, particularly through the inhibition of the mammalian target of the rapamycin (mTOR) pathway using compounds such as rapamycin, has demonstrated neuroprotective effects in the experimental models of neurodegeneration. Similarly, the activation of AMP-activated protein kinase (AMPK) through agents like metformin and statins has been proposed as an effective strategy to upregulate autophagic flux and mitigate neurodegenerative processes through the restoration of efficient cellular clearance mechanisms, the prevention of the accumulation of pathogenic protein aggregates, and the attenuation of neuroinflammation [10,11,12,13].

Given the pathological role of senescent cells in neurodegeneration, senolytic therapies have also emerged as a promising intervention. Agents like Navitoclax (ABT263), a well-characterized senolytic compound, selectively eliminate senescent astrocytes and endothelial cells, restoring neurovascular function and improving cognitive outcomes as demonstrated in several published models [14,15]. Targeting senescent cells may offer a novel strategy for slowing or preventing neurodegenerative diseases by reducing chronic inflammation and preserving neuronal function. As research continues, refining senolytic therapies, matching them to autophagic regulation, and assessing their long-term efficacy and safety will be crucial for their future clinical application [16,17].

This section will explore these therapeutic interventions for autophagy and senolysis, exploring their potential value as neuroprotectants.

### 3.1. Senolytic Mechanisms and Pharmacological Intervention for Neurovascular Protection

Cellular senescence contributes significantly to age-related neurodegenerative disorders through the establishment of chronic inflammation and neurovascular dysfunction. Senolytics are designed to selectively eliminate senescent cells, thereby attenuating the detrimental effects of SASP. These compounds target specific vulnerabilities in senescent cells while sparing healthy cells, potentially protecting against toxic build up, a hallmark of AD [15,18].

Navitoclax, a Bcl-2/Bcl-xL inhibitor, has demonstrated efficacy in targeting senescent endothelial cells within the cerebrovascular system. Research has shown that Navitoclax administration effectively eliminates senescent endothelial cells, resulting in the restoration of BBB integrity and enhanced cerebral blood flow. In models of chemotherapy-induced cognitive decline, a condition characterized by accelerated cellular senescence, Navitoclax treatment has yielded significant improvements in cognitive function, particularly in domains of learning and memory [19]. The restoration of neurovascular homeostasis appeared to be a primary mechanism underlying these cognitive benefits. For example, in a study involving aged (24 months old) C57BL/6 mice, treatment with Navitoclax improved neurovascular coupling responses and hippocampal-dependent learning and memory functions, associated with the elimination of senescent cells in the brain [20].

Further research has indicated that Navitoclax treatment can rejuvenate aged hematopoietic stem cells and muscle stem cells in mice, suggesting broader implications for age-related tissue degeneration [21]. While these preclinical findings are promising, clinical studies in humans are still in the early stages. Navitoclax is currently undergoing Phase III clinical trials primarily for cancer treatment, with some studies exploring its senolytic effects. However, comprehensive clinical data on its efficacy in mitigating cognitive decline are not yet available. Ongoing and future clinical trials will be essential to determine its safety and effectiveness in treating cognitive decline in humans [22].

Beyond endothelial cells, senescent astrocytes represent another critical target for senolytic intervention. Astrocytes, as essential components of the neurovascular unit, provide metabolic and structural support to neurons and contribute to BBB maintenance. However, senescent astrocytes secrete pro-inflammatory factors that disrupt these functions. The selective depletion of senescent astrocytes through senolytic compounds has been shown to reduce radiation-induced cognitive dysfunction, a condition associated with premature cellular senescence in the brain. In a study by Yabluchanskiy et al., mice that had undergone whole-brain irradiation to model radiation-induced cognitive dysfunction were treated with the senolytic compound Navitoclax (ABT-263). The treatment selectively targeted and eliminated senescent astrocytes and effectively restored neurovascular coupling responses, which are crucial for maintaining the dynamic relationship between cerebral blood flow and neuronal activity. Specifically, Navitoclax improved cerebrovascular reactivity in response to neuronal stimulation, thus enhancing blood flow to active brain regions. This restoration of neurovascular function was associated with significant improvements in hippocampal-dependent learning and memory tasks [23].

Targeting senescent cells to retain the stabilization of neurovascular unit function could benefit those at high risk of developing AD, and future research should focus on optimizing senolytic agents, refining their specificity, and assessing their long-term safety and efficacy in clinical applications [8,15,16].

### 3.2. Re-Normalization of Autophagy to Balance Senolysis, and Restoration of the NVU/BBB Microenvironment

As stated earlier, autophagy is a crucial cellular mechanism responsible for degrading and recycling damaged proteins and organelles, and a reduction in the efficiency of this process occurs concomitantly with the onset of AD [10,24].

One of the most studied autophagy enhancers is rapamycin, an mTOR inhibitor that enhances autophagic flux, thereby reducing Aβ accumulation and protecting neurons from degeneration. Studies have shown that rapamycin treatment can restore autophagy efficiency and promote neuronal survival in AD models [10,25]. Another compound, metformin, an AMPK activator, has been found to stimulate autophagy and enhance mitochondrial biogenesis. However, its effects in AD remain complex, as some studies suggest both neuroprotective and potentially adverse effects, warranting further investigation [26]. Additionally, statins, widely used for their cholesterol-lowering effects, have been shown to modulate autophagy pathways, potentially providing neuroprotection against AD-related pathology.

Below, we will describe the main protagonists, their mechanisms of action, potential synergy, and an overview of possible new combinational protective therapies.

#### 3.2.1. Rapamycin and mTOR Inhibition, a Cornerstone of Autophagic Modulation

Rapamycin, a macrolide compound originally isolated from Streptomyces hygroscopicus, has attracted significant interest for its potential neuroprotective properties. Its primary biological activity centers on the inhibition of the mechanistic target of mTOR, a serine/threonine kinase that, under nutrient-rich conditions, suppresses autophagy, a critical cellular housekeeping process involved in the degradation of damaged organelles and protein aggregates. Given the pivotal role of impaired proteostasis and defective autophagic clearance in the pathogenesis of AD, rapamycin–mTOR regulation may be considered a candidate for future therapeutic intervention [27].

**Figure 1:**
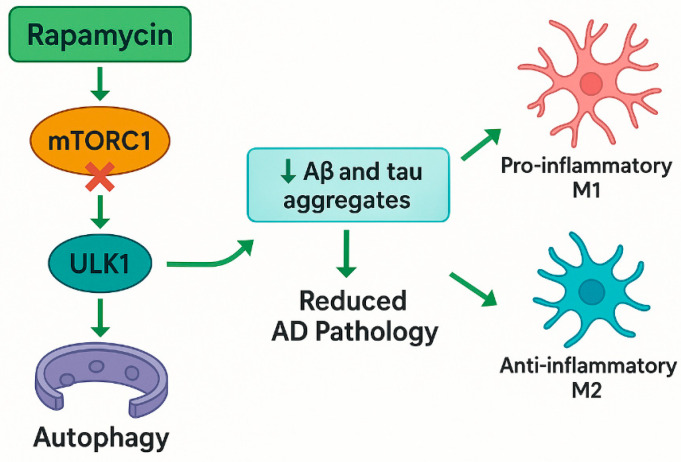
Schematic illustration of rapamycin’s neuroprotective mechanisms in AD, highlighting its direct inhibition of mTORC1 (independent of AMPK), the induction of autophagy via ULK-1 activation, reduction in amyloid and tau pathology, and the modulation of microglial polarization. Abbreviations: mTORC1—mammalian target of rapamycin complex 1; ULK1—unc-51-like autophagy-activating kinases 1; Aβ—amyloid beta; AD—Alzheimer’s disease.

Rapamycin inhibits the Mammalian Target of Rapamycin Complex 1 (mTORC1), thereby relieving suppression on the unc-51-like autophagy activating kinase 1 (ULK-1) complex and promoting autophagy initiation. Hwang et al. (2017) demonstrated that in oxygen–glucose deprivation (OGD)-induced rat CA1 hippocampal neurons, rapamycin-mediated mTORC1 inhibition led to ULK-1 phosphorylation at Ser757, triggering autophagosome formation and enhancing autophagic flux. Additionally, rapamycin increased the phosphorylation of ATG13 (Ser318), further facilitating autophagosome biogenesis. Beyond direct autophagy activation, rapamycin also modulates the mitogen-activated protein kinase/Extracellular Signal-Regulated (MEK/ERK) pathway, upregulating Beclin-1, a critical autophagy protein, and Noxa, a pro-apoptotic molecule that disrupts the inhibitory interaction between Mcl-1 and Beclin-1, thus favoring autophagic progression. The downregulation of p62, a marker of defective autophagy, also supported the efficient turnover of toxic protein aggregates [28] (see Figure 1).

Preclinical studies demonstrate that rapamycin-induced mTOR inhibition reduces Aβ plaques and hyperphosphorylated tau in AD models, improving synaptic plasticity and cognitive function. Summarized eloquently by Carosi et al. (2023), they noted that while the early administration of rapamycin in transgenic mice promotes autophagy and can prevent Aβ and tau accumulation, its effectiveness was highly dependent on timing and lysosomal function. In models with compromised lysosomal degradation, rapamycin often worsened pathology by causing autophagic stress, leading to the accumulation rather than clearance of toxic proteins. Notably, rapamycin reduced tau aggregates in early-stage models but failed in later stages or in models with impaired lysosomal capacity, such as 5xFAD mice. These findings suggest that enhancing autophagy through mTOR inhibition may be beneficial only when lysosomal function is intact, highlighting the need for patient-specific or stage-specific approaches in potential AD treatments [29].

Yun et al. (2024), using a murine model of AD (Aβ injection), demonstrated the modulation of the AMPK/mTOR signaling pathway through FoxG1, which not only enhanced autophagy but also redirected microglial polarization from pro-inflammatory M1 to anti-inflammatory M2 phenotypes, thereby creating a neuroprotective microenvironment conducive to cellular recovery and tissue homeostasis (Figure 1) [30]. Rapamycin also mitigated neurodegeneration in axonal injury models. Optic nerve axotomy experiments revealed that rapamycin enhanced autophagy in retinal ganglion cells, reducing neuronal death [31]. Similarly, in chemotherapy-induced cognitive decline models, rapamycin analogs like Navitoclax eliminate senescent cells, restoring neurovascular coupling and cognitive function [20,32].

However, its immunosuppressive effects at high doses and long-term safety profile in humans require careful evaluation. Clinical trials exploring low-dose, intermittent rapamycin regimens (e.g., 5–10 mg weekly) are underway, inspired by its potential to mimic fasting-induced autophagy without metabolic stress [33]. Although there are, to date, no completed trials, two early-phase clinical trials are currently investigating rapamycin as a potential treatment for AD. A Phase 2 randomized, placebo-controlled trial, initiated in August 2021, is evaluating the safety and cognitive effects of daily oral rapamycin over one year in 40 individuals aged 55–89 with mild cognitive impairment or early-stage AD, with outcomes including cognitive performance, daily function, and brain imaging markers. Separately, the ERAP Phase IIa study is an open-label, six-month trial in 15 patients with early-stage AD, administering a weekly 7 mg dose of rapamycin to assess changes in cerebral glucose metabolism via [18F] FDG-PET, along with cognitive and cerebrospinal fluid biomarkers. Both trials aim to determine rapamycin’s potential as a disease-modifying therapy in early AD.

#### 3.2.2. Metformin (AMPK Activator) Could Be a Multifaceted Therapeutic Agent in Autophagy, AD, and Anti-Aging

Metformin, a first-line therapy for type 2 diabetes, activates AMPK, a cellular energy sensor that enhances autophagy and mitochondrial biogenesis. Metformin also activates Sirtuin 1 (SIRT1), a key regulator of cellular energy balance and stress resistance. SIRT1 further promotes neuroprotection by modulating transcription factors like Peroxisome Proliferator-Activated Receptor Gamma Coactivator 1-alpha (PGC-1α), which regulate mitochondrial function and antioxidant defenses [34,35].

Metformin has demonstrated remarkable anti-aging effects across various models. A study by Yang et al. (2024) over 40 months, on cynomolgus monkeys, showed that chronic metformin administration decelerated aging biomarkers, including transcriptomic and epigenomic alterations associated with brain aging. Notably, metformin preserved brain structure and function by activating nuclear factor erythroid 2-related factor 2 (Nrf2)-mediated antioxidative pathways [36]. Previously, some insights into the mechanisms associated with this finding were suggested by Chung et al. (2015), where age-related cognitive dysfunction was protected against by increased mitochondrial biogenesis and reduced oxidative stress through AMPK/SIRT1 activation in human neural stem cells treated with AGEs. These mechanisms improve neuronal resilience against metabolic stressors, positioning metformin as a geroprotective agent [37].

The primary mechanism through which metformin influences autophagy is the activation of AMPK, a cellular energy sensor that responds to metabolic stress. Benito-Cuesta et al. (2020) demonstrated in APP/PSEN1 transgenic mice and primary cerebellar neurons that mTORC1 inhibition via rapamycin reduced Aβ by 50% through autophagy induction, validated by Western blot (microtubule-associated protein 1 light chain 3 (LC3) LC-II/LC3-I ratio) and immunofluorescence [38]. García-Juan et al. (2024) extended these findings using APP/PSEN1 astrocytes, confirming mTORC1 inhibition’s role in Aβ clearance but demonstrating that AMPK activation (via AICAR/metformin) paradoxically reduced autophagy flux while lowering Aβ secretion through AMPK-independent pathways [39].

In gastric cancer cells, metformin activated AMPK, inhibited mTOR, and upregulated LC3/Beclin-1, culminating in an increased autophagic flux [40]. Mechanistically, AMPK activation blocked the mTORC1-mediated suppression of autophagy initiation complexes (ULK1/ATG13), promoting Aβ/tau clearance. However, cell-type specificity emerged: AMPK activation upregulated autophagy genes in cancer cells but reduced autophagy in astrocytes despite Aβ reduction. These findings underscore AMPK’s dual role, enhancing proteostasis in neurons while exerting divergent effects in glial cells. The studies collectively support the concept of mTORC1 inhibition as a potential strategy for Aβ clearance, whereas AMPK’s therapeutic potential in AD requires cell-contextual precision. Chronic AMPK activation may mitigate AD pathology, but its application must account for cell-specific autophagy regulation to avoid unintended effects.

Xu et al. (2021) demonstrated that metformin activates chaperone-mediated autophagy (CMA) through the TAK1-IKKα/β signaling pathway, a mechanism distinct from its AMPK-dependent macroautophagy induction. Using HEK293T cells and APP/PS1 transgenic mice, the study revealed that metformin phosphorylates Hsc70 at Ser85 via TAK1-IKKα/β, enhancing its binding to CMA substrates like the amyloid precursor protein (APP). This selective degradation of APP reduced Aβ levels by 55% in mouse models and was shown by immunohistochemistry and ELISA. Behavioral tests, including the Morris water maze, confirmed improved cognitive performance in metformin-treated APP/PS1 mice. Concurrently, metformin activated macroautophagy via AMPK, synergistically enhancing proteostasis by clearing protein aggregates. The dual activation of CMA and macroautophagy underscored metformin’s unique ability to target multiple proteolytic pathways, offering a multimodal approach to AD therapy. These findings were validated in human iPSC-derived neurons, where metformin reduced tau phosphorylation and Aβ secretion [34].

**Figure 2:**
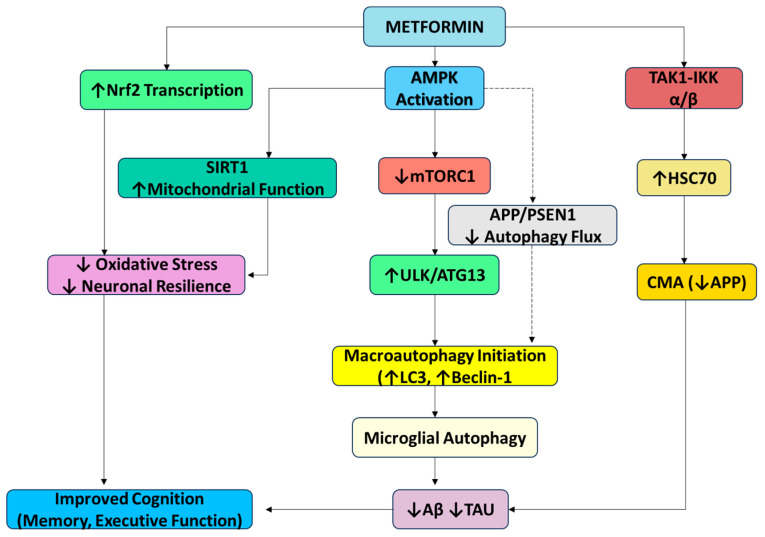
Metformin-mediated modulation of autophagy pathways: This diagram summarizes the key signaling mechanisms through which metformin modulates both macroautophagy and CMA. Metformin activates AMPK, leading to mTORC1 inhibition and the upregulation of ULK1/ATG13, thereby enhancing autophagic flux via LC3 and Beclin-1. Independently, it activates CMA through the TAK1–IKKα/β pathway and Hsc70 phosphorylation. Additional neuroprotective effects include Nrf2-mediated antioxidant defense and improved mitochondrial function via SIRT1. While microglial autophagy supports Aβ and tau clearance, AMPK activation in astrocytes may paradoxically reduce autophagy, highlighting cell-type specificity. Inhibition (red); activation (green). Abbreviations: mTOR—mammalian target of rapamycin; BDNF—brain-derived neurotrophic factor; Aβ—amyloid beta; AMPK—AMP-activated protein kinase; ULK1—Unc-51-like autophagy-activating kinases; SIRT-1—silent mating-type information regulation 2 homolog; APP—amyloid precursor protein; PSEN-1—presenilin-1; TAK1—transforming growth factor β-activated kinase 1; HSC70—Heat Shock Cognate protein 70; ATG13—autophagy-related protein 13; Iκκ—I kappa B kinase.

Benito-Cuesta et al. (2023) and Garcia-Juan et al. (2023) examined metformin’s role in autophagy regulation, revealing limitations in aging and disease. Benito-Cuesta et al. used aged neuronal cultures and APP/PS1 mice, showing that while metformin promoted autophagy in younger cells, chronic use in aged cells impaired lysosomal function and exacerbated protein aggregation due to mitochondrial ROS overproduction. Metformin activated AMPK but paradoxically suppressed autophagy flux in LPS-stimulated astrocytes and APP/PS1 mice by impairing lysosomal acidification. These studies emphasized the age- and cell-specific effects of metformin, where its benefits in reducing Aβ and tau aggregates are hindered by lysosomal dysfunction in aged or metabolically compromised cells [38]. Garcia-Juan et al. (2023) also highlighted that while metformin activates AMPK, this alone does not consistently enhance the autophagic clearance of Aβ in astrocytes, indicating that the modulation of autophagy pathways is context-specific. This suggests that AMPK activation may not be sufficient to drive the effective autophagic clearance of Aβ in astrocytes, particularly under conditions of metabolic stress or aging (see Figure 2). As a result, further investigation is needed into other mechanisms that could modulate astrocyte-mediated autophagy, such as alternative signaling pathways, mitochondrial dynamics, or interactions with other cellular components like the endoplasmic reticulum and lysosomal networks, to better understand how to optimize autophagic flux and enhance Aβ clearance in these cells [39].

Preclinical studies, such as those by Chen et al. (2021) in APP/PS1 transgenic mice, demonstrated that metformin reduces amyloid plaque deposition and tau pathology by activating microglial autophagy, facilitating the phagocytosis of Aβ and tau aggregates; these findings were corroborated by Xu et al. (2021), who showed that metformin activates chaperone-mediated autophagy, selectively degrading APP and reducing tau aggregation [34,41]. Although these preclinical findings are promising and suggest metformin’s potential as a multi-target therapeutic for AD by modulating AMPK/mTOR/SIRT1 pathways [42], clinical evidence remains mixed, and observational studies suggest potential cognitive benefits but highlight conflicting data regarding its long-term impact on dementia prevention, emphasizing the need for large, long-term randomized trials like the TAME study to clarify metformin’s role in dementia prevention [43].

Recent clinical and meta-analytical studies have reported the heterogeneous effects of metformin on cognitive decline and AD risk. A 2021 meta-analysis of ten observational studies (229,110 participants) found no significant association between metformin use and AD incidence overall, but subgroup analysis revealed an increased AD risk among Asian patients (OR = 1.71) [44,45]. Additional meta-analyses and epidemiological studies indicated that metformin may slow cognitive decline in diabetic populations, with some trials reporting reduced dementia risk and improved executive function, particularly with long-term use (>6 years) [46,47,48]. However, these neuroprotective effects were not consistently observed for AD risk, and some studies even noted increased AD risk in specific subgroups, such as those with certain APOE genotypes or longer diabetes duration [49]. Mechanistically, metformin’s cognitive benefits have been attributed to improved insulin sensitivity, reduced vascular burden, and enhanced mitochondrial function, though these pathways may interact with genetic and metabolic factors. Notably, clinical outcomes varied according to treatment duration, dose, and patient characteristics, highlighting the need for individualized approaches and further randomized trials. Hence, current evidence suggests that while metformin may confer cognitive benefits in some diabetic populations, its impact on AD risk remains inconclusive and potentially context dependent.

While rapamycin and metformin both regulate autophagy and exhibit promise in aging and neurodegenerative disorders, they operate through fundamentally distinct mechanisms. Rapamycin acts primarily by directly inhibiting the mechanistic target of mTORC1, thereby promoting autophagy under nutrient-rich conditions. In contrast, metformin modulates autophagy indirectly, mainly through the activation of AMPK, a cellular energy sensor, and also engages additional pathways such as CMA and oxidative stress regulation. These divergent pathways result in distinct cellular outcomes, particularly in the context of AD, where cell-type specificity and the timing of autophagic modulation can critically affect therapeutic outcomes.

#### 3.2.3. PDE5 Inhibitors as Novel Therapeutics in AD; Mechanisms and Evidence

PDE5 inhibitors, including sildenafil and tadalafil, are clinically approved for erectile dysfunction (ED) and pulmonary arterial hypertension (PAH). However, through enhancing nitric oxide (NO)–cyclic guanosine monophosphate (cGMP) signaling, PDE5 inhibitors improve vascular compliance, cerebral blood flow, and mitochondrial function, addressing core pathways implicated in AD and cognitive decline [50].

Kang et al. (2022) investigated the therapeutic potential of mirodenafil, a BBB-penetrant PDE5 inhibitor, in AD. Using SH-SY5Y cells and APP-C105 transgenic mice, the study demonstrated that mirodenafil enhances the autophagic clearance of Aβ via AMPK activation, independently of mTOR signaling. Western blot analysis confirmed increased phosphorylated AMPK and autophagy markers, whilst behavioral assessments, including the Morris water maze and passive avoidance tests, showed improved cognitive performance in treated mice. Mirodenafil also reduced the levels of Aβ and phosphorylated tau, acting through the cGMP/PKG/cAMP Response Element-Binding Protein (CREB) pathway and inhibiting GSK-3β, a kinase involved in tau pathology. Additionally, it modulated glucocorticoid receptor activity by inhibiting its nuclear translocation and dimerization—an effect unique to mirodenafil among PDE5 inhibitors. The compound further activated Wnt/β-catenin signaling by suppressing the Wnt antagonist Dkk-1 and promoted autophagy–lysosomal function. These findings suggested that mirodenafil exerts multi-targeted neuroprotective effects, positioning it as a promising polypharmacological agent for AD therapy [51].

**Figure 3:**
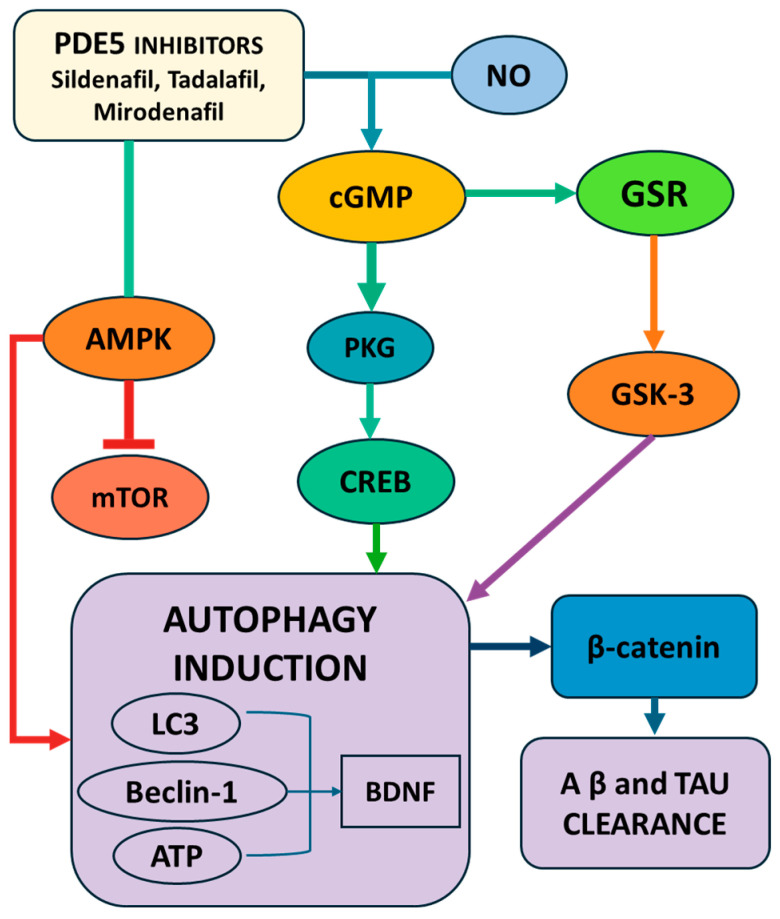
Schematic representation of the molecular pathways by which PDE5 inhibitors (sildenafil, tadalafil, mirodenafil) promote autophagy induction and neuroprotection. PDE5 inhibition increases cGMP levels, activating PKG and downstream CREB signaling, which upregulates autophagy-related proteins (LC3, Beclin-1, BDNF) and facilitates Aβ and tau clearance. The parallel modulation of the AMPK/mTOR and GSK-3/β-catenin pathways further enhances autophagy and reduces neurodegenerative protein accumulation. Abbreviations: LC3—microtubule-associated proteins 1A/1B light chain 3; mTOR—mammalian target of rapamycin; BDNF—brain-derived neurotrophic factor; Aβ—amyloid beta; AMPK—AMP-activated protein kinase; CREB—cAMP Response Element-Binding Protein; PKG—protein kinase G; ATP—adenosine tri-phosphate.

Sildenafil, also a PDE5 inhibitor, regulated autophagy in an experimental auto-immune encephalomyelitis (EAE) mouse model of multiple sclerosis. Here, immunoblotting assessed the expression levels of autophagy markers LC3, Beclin-1, and ATG5, along with mTOR activity and p-AMPK levels, and showed that sildenafil treatment increased autophagy markers while decreasing mTOR levels and enhancing p-AMPK activity. These findings suggest that sildenafil activates autophagy via the endothelial Nitric Oxide Synthase (eNOS)-NO-AMPK-mTOR-LC3-Beclin1-ATG5 pathway, indicating potential neuroprotective effects in neurodegenerative diseases like multiple sclerosis (Figure 3). Sildenafil improved motor function and reduced clinical scores in EAE mice, enhanced autophagy to clear protein aggregates and mitigate neurodegeneration, and restored p-CREB and brain-derived neurotrophic factor (BDNF) levels to promote synaptic plasticity, highlighting sildenafil’s dual role in reducing neuroinflammation and enhancing proteostasis through mTOR-independent AMPK activation [52].

In vivo studies, using tadalafil-treated (8 weeks) diabetic db/db mice, demonstrated enhanced NO levels, activated SIRT1, and increased PGC-1α expression, improving mitochondrial oxidative phosphorylation (OXPHOS) and reducing ROS generation in the heart. The results showed improved heart function, enhanced eNOS, Protein Kinase B (Akt), and AMPK phosphorylation, and protection against mitochondrial dysfunction in diabetic mice, highlighting tadalafil’s potential to activate NO-induced SIRT1-PGC-1α signaling [53].

Together, these studies highlight the diverse mechanisms by which PDE5 inhibitors, including mirodenafil, sildenafil, and tadalafil, modulate autophagy, inflammation, and mitochondrial function, offering potential therapeutic avenues for neurodegenerative and metabolic diseases.

Preclinical studies using APP/PS1 transgenic mice, treated with sildenafil intra-peritoneally at a dose of 3 mg/kg, examined electrophysiological recordings from hippocampal slices to assess long-term potentiation (LTP), a key marker of synaptic plasticity, alongside behavioral tests such as contextual fear conditioning and the radial arm water maze to evaluate memory and learning. The biochemical analyses of cortical homogenates revealed that sildenafil reduced Aβ levels by 30%. Mechanistically, these benefits were linked to the activation of the cGMP-PKG-CREB signaling pathway, which plays a critical role in synaptic function and memory formation. The study concluded that sildenafil improves synaptic plasticity and cognitive performance in AD mice, while simultaneously reducing Aβ burden, supporting its potential as a therapeutic strategy targeting both the molecular and functional aspects of AD pathology [54].

Abouelmagd and Abdelmeseh et al. (2024) performed a meta-analysis of six observational studies (N = 245,095) to evaluate the association between PDE5 inhibitor use and AD risk. Utilizing pooled hazard ratios (HRs) and addressing high heterogeneity (I^2^ = 99%), the analysis revealed a 47% reduction in AD risk (HR = 0.53) among PDE5 inhibitor users. Subgroup analyses highlighted stronger effects in sildenafil users (54% risk reduction) and women (47% reduction). Proposed mechanisms included enhanced cerebral perfusion, Aβ clearance via LRP1 upregulation, and preserved neurovascular coupling, alongside PPARγ/NF-κB pathway modulation to suppress neuroinflammation. However, short-term tadalafil trials showed neutral cognitive outcomes, potentially due to suboptimal dosing or heterogeneous patient populations. The authors concluded that chronic PDE5 inhibition may delay AD onset but emphasized the need for optimized dosing strategies and biomarker-driven clinical trials to validate these findings and refine therapeutic protocols [55].

A large-scale epidemiological study recently analyzed insurance claim data from over 7 million individuals to evaluate sildenafil’s association with AD risk. Using retrospective case–control analyses, the researchers compared sildenafil users to non-users, adjusting for confounders (age, sex, race, comorbidities) via propensity score stratification. The study identified a 31% reduction in AD hazard among sildenafil users (HR = 0.69, 95% CI: 0.57–0.83), with mechanistic insights derived from induced pluripotent stem cell (iPSC)-based neuron models. These models demonstrated sildenafil’s capacity to reduce tau hyperphosphorylation (pTau181 and pTau205) and enhance neurite growth, implicating cGMP-PKG-CREB signaling and NF-κB suppression as key neuroprotective pathways. Their findings suggested that sildenafil mitigated AD pathology primarily by enhancing synaptic plasticity and Aβ clearance. While the observational design limited causal inference, the study underscored sildenafil’s potential as a repurposed therapeutic for AD, bridging computational drug discovery with preclinical validation. The authors emphasized the necessity of randomized clinical trials to confirm efficacy and optimize therapeutic protocols [56].

Together, preclinical and clinical evidence support the multimodal potential of PDE5 inhibitors in neurodegenerative and metabolic diseases. Through convergent mechanisms (autophagy induction, anti-inflammatory effects, improved mitochondrial function, and vascular protection), these agents may be useful adjuncts in AD therapy and healthy brain aging. However, further randomized clinical trials and precision medicine approaches are essential to validate efficacy, optimize dosing, and identify responsive patient populations.

Unlike either metformin or rapamycin, PDE5 inhibitors activate autophagy primarily through cGMP-PKG-NO-eNOS-AMPK signaling, with evidence of mTOR-independent autophagy induction (especially in the case of mirodenafil). Their mechanisms are multi-targeted, also involving CREB/BDNF signaling, the inhibition of GSK-3β, and Wnt/β-catenin activation, positioning them uniquely as neurotrophic and vasoprotective agents.

#### 3.2.4. Benzimidazole Derivatives: Repurposed for Autophagy and Senescence Modulation in AD

Benzimidazole derivatives, traditionally utilized as anthelmintics, may have additional therapeutic potential beyond parasitic infections, with recent studies characterizing a role in modulating apoptosis and autophagy, making them candidates for repurposing in oncology and neurodegeneration. Benzimidazole derivatives (e.g., flubendazole (FLBZ) and albendazole) may have therapeutic potential in modulating neurodegeneration and aging, particularly since they have demonstrated the ability to induce lysosomal clustering, enhance autophagy, and reduce neuroinflammation.

Benzimidazole derivatives, such as FLBZ, induce autophagy through mTOR-independent pathways, avoiding metabolic disruptions associated with conventional mTOR inhibitors. Zhang et al. (2021) screened over 30 compounds for their ability to induce autophagy in primary human retinal pigment epithelial (RPE) cells without directly inhibiting mTOR. FLBZ treatment led to the reduced secretion of apolipoproteins associated with drusen (yellow deposits of lipids and protein accumulating under the retina) formation, the increased production of β-hydroxybutyrate, the decreased accumulation of lipofuscin, and alleviated lipofuscin-induced cellular senescence and tight-junction disruption in RPE cells. Therefore, FLBZ may offer therapeutic benefits for dry age-related macular degeneration, promoting autophagy without the adverse effects associated with chronic mTOR inhibition (e.g., hyperlipidemia, insulin resistance, muscle wasting, etc.), and this could potentially be applied to AD protection [57].

In order to examine the mechanistic pathways in more detail, Date et al. (2024) used high-throughput screening in SH-SY5Y human neuroblastoma cells to show that albendazole, a benzimidazole derivative, activated the JIP4–TRPML1 pathway, promoting lysosomal clustering at the microtubule-organizing center and enhancing autophagosome–lysosome fusion. This was sufficient to facilitate the clearance of α-synuclein and hyperphosphorylated tau aggregates. At low doses, albendazole selectively modulated peripheral microtubules, whilst at higher doses, it disrupted tubulin globally. Unlike rapamycin, it preserved mTOR signaling, whilst co-treatment with mTOR inhibitors increased autophagic flux [58].

**Figure 4:**
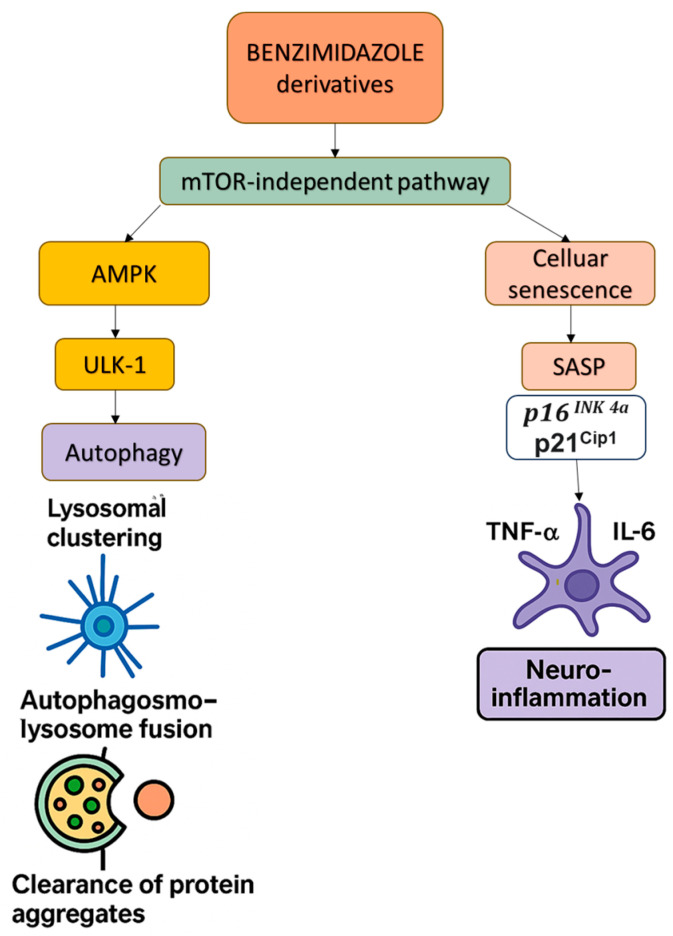
This diagram illustrates the mTOR-independent modulation of autophagy and cellular senescence by benzimidazole derivatives (e.g., FLBZ and albendazole). These compounds promote lysosomal clustering and autophagosome–lysosome fusion, facilitating the clearance of protein aggregates via AMPK and ULK-1 activation. Simultaneously, they reduce cellular senescence and SASP-driven neuroinflammation by suppressing key pro-inflammatory cytokines (TNF-α and IL-6), highlighting their therapeutic potential in neurodegenerative diseases. Abbreviations: mTOR—mammalian target of rapamycin; SASP—senescence-associated secretory phenotype; TNF-α—Tumor Necrosis Factor alpha; IL-6—Interleukin-6.

Cellular senescence is primarily driven by p16INK4a/p21CIP1 activation and SASP-mediated inflammation, and this has been shown to exacerbate neurodegeneration by promoting BBB dysfunction and neuroinflammation. Zhang et al. (2021) demonstrated that FLBZ reduced senescence in RPE cells by enhancing autophagy flux, restoring tight-junction integrity, and alleviating lipofuscin accumulation. FLBZ achieved this by promoting lysosomal clustering without inhibiting mTOR, thereby preserving metabolic balance [57]. In addition, Ullah et al. (2022) and others showed that benzimidazole derivatives suppressed SASP factors (e.g., TNF-α and IL-6) and NF-κB activation in ethanol-induced rat (Sprague Dawley) neurodegeneration models, associated with shifting microglia to anti-inflammatory M2 phenotypes via NLRP3 inflammasome inhibition [59,60]. These findings aligned with Yabluchanskiy et al. (2023), who highlighted SASP factors as the key drivers of neurotoxic glial activation using transgenic p16-3MR mice. Together, these studies support a possible role for benzimidazoles as senomorphic agents capable of mitigating age-related neurodegeneration through dual mechanisms: autophagy-mediated aggregate clearance and SASP suppression [23] (Figure 4).

Benzimidazole derivatives have been created as senolytics targeting Bcl-2/Bcl-xL in various colorectal cancer cell lines. Using molecular docking and in vitro assays, it was demonstrated that these compounds induced apoptosis and increased autophagy. The derivatives disrupted apoptosis resistance similarly to Navitoclax. Their dual action suggested, therefore, potential synergistic effects modulating/protecting against neurodegeneration [61]. Similarly, Cevik et al. (2019) synthesized a series of benzimidazole–triazole derivatives and evaluated their potential as acetylcholinesterase (AChE) inhibitors. Among these, compounds 3d and 3h (characterized by 3,4-dihydroxy substitutions on the phenyl ring and 5(6)-chloro substitutions on the benzimidazole ring) demonstrated potent nanomolar inhibitory activity, with IC₅₀ values of 31.9 ± 0.1 nM and 29.5 ± 1.2 nM, respectively. Using Ellman’s colorimetric method and kinetic analyses, both compounds exhibited mixed inhibition mechanisms. Molecular docking confirmed strong interactions at both the catalytic active site and the peripheral anionic site of AChE, suggesting dual-site binding [62].

Further studies developing benzimidazole–thiazole hybrid compounds indicated potential cholinesterase inhibition in vitro, showing dual inhibitory activity against AChE and butyrylcholinesterase (BChE), with sub-micromolar to low micromolar IC₅₀ values. Computational pharmacokinetic simulations predicted favorable BBB permeability. In vivo studies in AD mouse models demonstrated that these hybrids reduced Aβ plaque accumulation and improved cognitive performance without notable adverse effects. Together, these findings indicate the potential use of benzimidazole derivatives as multi-target agents in AD, combining potent enzyme inhibition with neuroprotective and drug-like properties [63,64].

In studies using the APP/PS1 transgenic mouse model of AD, FLBZ was shown to reduce Aβ plaques and attenuate tau hyperphosphorylation. FLBZ activated autophagy through the PPARγ pathway, facilitating the clearance of Aβ plaques, and also inhibited Glycogen Synthase Kinase 3 Beta (GSK3β), which reduced tau hyperphosphorylation [65]. Additionally, co-treatment with Torin1, an mTOR inhibitor, further enhanced the clearance of protein aggregates by ‘boosting’ autophagic flux, indicating a possible synergistic approach for therapies [66]. One additional finding of note is the interest in identifying glutaminyl cyclase inhibitors, which appear to block Aβ aggregation in vivo. Here, the derivatives of benzimidazole, such as PQ912, appeared safe and effective in both in vitro studies all the way to Phase-2a trials [67]. This could be an additional benefit when considering future combinational and synergistic approaches to therapy.

To date, there is no other direct clinical evidence published referring to the impact of benzimidazole class compounds on neurodegeneration, cognition, or AD in humans.

#### 3.2.5. Acetylsalicylic Acid (ASA) in Autophagy, Senescence, and Neurodegeneration: Mechanisms of Action and Therapeutic Potential

ASA, best known for its anti-inflammatory and antiplatelet activity, has emerged as a multifaceted modulator of pathways critical to brain aging and neurodegeneration. Beyond its cardiovascular applications, ASA influences autophagy, mitigates cellular senescence, and suppresses neuroinflammation, key mechanisms implicated in AD and age-related cognitive decline [68].

ASA induces autophagy activation via pro-resolving (restoring homeostasis by negating inflammation) and cyclooxygenase-2 (COX-2) pathways: it triggers the production of specialized pro-resolving mediators (SPMs; small lipid-based molecules), particularly aspirin-triggered resolvin D1 (AT-RvD1), in a rat neuropathic pain model of L5-6 spinal nerve ligation. SPMs also upregulated LC3B and Beclin-1, as shown in a TNF-α-induced microglial culture model, driving the autophagosome formation and clearance of Aβ and phosphorylated tau. Concurrently, AT-RvD1 suppressed NLRP3 inflammasome activation in microglia, reducing neurotoxic cytokine release while promoting the lysosomal degradation of pathological aggregates [69]. ASA’s non-selective inhibition of COX-1/2 reduces prostaglandin-driven neuroinflammation, indirectly influencing mTOR activity. While chronic COX-2 suppression may impair synaptic plasticity, low-dose ASA preserves hippocampal function by balancing mTOR-dependent autophagy and prostaglandin-mediated synaptic signaling in addition to a reduction in the activity of AChE [70].

In addition, ASA also causes the suppression of cellular senescence through the mitigation of SASP by inhibiting NF-κB, a master regulator of pro-inflammatory cytokines (IL-6 and TNF-α). This reduces oxidative stress and mitochondrial dysfunction, key triggers of neuronal senescence [71]. Studies in hypoxic neonatal mice and in microglial cell models utilizing PPARγ inhibitors have also shown that enhanced PPARγ signaling effectively counteracts neuroinflammation, preserving BBB integrity and synaptic function by the suppression of the high mobility group box 1 protein (HMGB1)/NF-κB signaling pathways [72].

**Figure 5:**
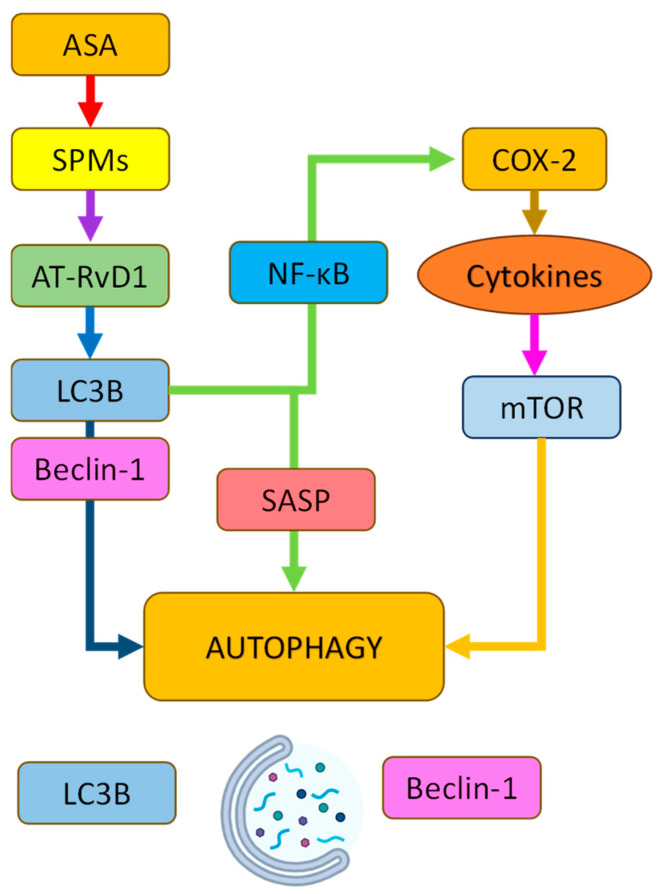
Schematic representation of the signaling pathways by which acetylsalicylic acid (ASA) modulates autophagy. ASA promotes the production of specialized pro-resolving mediators (SPMs), particularly RvD1, which enhances the expression of autophagy-related proteins LC3B and Beclin-1 while suppressing NLRP3 inflammasome and SASP via NF-κB inhibition. ASA also reduces COX-2-mediated neuroinflammation, indirectly modulating mTOR activity. Together, these pathways converge to activate autophagy and promote the clearance of pathological aggregates. *Abbreviations:* LC3B—microtubule-associated proteins 1A/1B light chain 3B; RvD1—resolvin D1; NF-κB—nuclear factor kappa B; COX-2—cyclooxygenase-2; mTOR—mammalian target of rapamycin; SASP—senescence-associated secretory phenotype.

Although direct evidence linking ASA to the clearance of senescent astrocytes is limited, emerging studies suggest that it may exert senomorphic effects in the brain by suppressing neuroinflammation and reducing SASP factors via the inhibition of NF-κB and mitogen-activated protein kinase (MAPK) pathways. Research has shown that ASA can attenuate glial activation, including that of astrocytes and microglia, in aging models, potentially lowering the burden of dysfunctional glial cells. While its role as a true senolytic in the central nervous system remains unconfirmed, ASA’s anti-inflammatory and SASP-modulating actions position it as a promising candidate for mitigating glial-driven neurodegeneration [73] (Figure 5).

Epidemiological studies report mixed results; in general, long-term low-dose ASA use correlates with reduced dementia risk in some cohorts but shows neutral effects in others [74]. This may be partially due to differences in genetic factors (e.g., apolipoprotein E4 (APOE4) status) and baseline inflammation levels, which could influence efficacy, necessitating personalized dosing strategies. The recent “Aspirin in Reducing Events in the Elderly (ASPREE)” was a double-blind, placebo-controlled trial that used low-dose aspirin to treat almost 20,000 individuals over 5 years, concluding that acetylsalicylic acid did not protect against cognitive decline or AD [75]; however, in combination with other substances, this may not hold true.

Studies show that low-dose ASA preserves LTP by moderating COX-2 activity, whereas high doses disrupt prostaglandin-dependent synaptic signaling. Cognitive performance, synaptic plasticity, and molecular markers of inflammation were assessed using both in vivo and in vitro models, in ASA-treated versus control groups. ASA administration improved LTP in the hippocampus and enhanced memory performance in behavioral tasks. It also significantly reduced COX-2 expression and the levels of pro-inflammatory prostaglandins. These findings suggest that low-dose ASA may preserve cognitive function by attenuating COX-2-mediated neuroinflammation and maintaining synaptic integrity [74,76].

ASA promotes Aβ clearance in AD mouse models by stimulating lysosomal biogenesis via the activation of the PPARα–TFEB pathway. This enhancement of the autophagy–lysosome system led to a significant reduction in Aβ plaque burden. Although mTOR was not the primary focus, its regulatory relationship with TFEB suggests indirect involvement. ASA-treated mice also showed improved cognitive performance. These findings highlight ASA’s potential in modulating protein clearance pathways relevant to neurodegeneration (reviewed by [77]). A summary comparison of these compounds is presented below in Table 1.

#### 3.2.6. A Note About Statins

Statins can modulate autophagy but may be inherently toxic, actually promoting neurodegenerative disease. Statins, widely prescribed for hyperlipidemia, have demonstrated complex roles in autophagy regulation and neuroprotection during brain aging. Some preclinical studies revealed that statins induce autophagy through AMPK/mTOR pathway activation, enhancing the clearance of Aβ and tau aggregates in AD models (e.g., simvastatin reduced neuroinflammation and oxidative stress in ischemic stroke models by upregulating pAMPK/LC3B/LAMP2 signaling, promoting autophagolysosome-mediated mitophagy) [78]. However, other studies showed that statins inhibited autophagy maturation by depleting geranylgeranyl pyrophosphate (GGPP), a mevalonate pathway derivative critical for autophagosome–lysosome fusion, leading to autophagic vacuole accumulation and neuronal toxicity in amyotrophic lateral sclerosis (ALS) models [79].

Human observational studies have reported mixed outcomes. A 2021 meta-analysis of 36 studies found statins associated with a 20% lower dementia risk (OR = 0.80) and a 32% reduced AD risk (OR = 0.68), particularly with high-potency statins [80]. However, randomized trials highlighted cognitive side effects, including memory deficits, in APOE ε4 carriers and elderly populations, linked to statins’ disruption of cholesterol-dependent synaptic plasticity and mitochondrial dysfunction in insulin-resistant brains [81]. Age-stratified analyses suggested that statins are neuroprotective only when initiated early in AD pathogenesis, before significant Aβ accumulation [82].

Therapeutic hesitancy also arises from statins’ narrow therapeutic window. While low doses enhance autophagy and reduce neuroinflammation, chronic use exacerbates lysosomal dysfunction in aged cells, worsening protein aggregation [83]. Additionally, statins’ systemic suppression of co-enzyme Q10 and vitamin D synthesis may accelerate mitochondrial decline in pre-disposed individuals. Current evidence therefore advocates a preference not to include statins in new combinational therapeutic approaches in AD [84].

## 4. Synergistic and Overlapping Signaling Molecules in Autophagic Modulation: A Case for Therapeutic Combination

The interplay between mTOR, ASA, PDE5 inhibitors, metformin, and benzimidazole derivatives converges on shared molecular hubs that regulate autophagy, inflammation, and cellular senescence. Central to this network is the AMPK/mTOR axis, where metformin and PDE5 inhibitors activate AMPK, indirectly inhibiting mTORC1 via raptor phosphorylation to promote autophagy, while rapamycin directly blocks mTORC1 to relieve its suppression of ULK-1/ATG13 and initiate autophagosome formation. This axis is further modulated by ASA, which inhibits mTOR through both AMPK-dependent and independent pathways, enhancing autophagic flux [85].

The Beclin-1/LC3 autophagy core serves as a critical convergence point; rapamycin, PDE5 inhibitors, and ASA upregulate Beclin-1, facilitating autophagosome assembly, while ASA (via AT-RvD1) and PDE5 inhibitors enhance LC3 lipidation, a marker of autophagosome maturation. These agents also intersect at inflammatory pathways, with rapamycin and ASA suppressing NF-κB and its pro-inflammatory mediators (TNF-α, IL-1β, IL-6), thereby reducing neuroinflammation. Similarly, benzimidazoles and ASA mitigate SASP by inhibiting p16INK4a/p21CIP1 and SASP cytokines, countering age-related cellular dysfunction.

Lysosomal coordination could be achieved through complementary mechanisms: benzimidazoles (e.g., FLBZ) promote lysosomal clustering via JIP4-TRPML1, while ASA enhances lysosomal degradation through AT-SPMs, synergizing with mTOR inhibition to clear pathological aggregates like Aβ and tau1. Metabolically, metformin and PDE5 inhibitors activate SIRT1/PGC-1α, improving mitochondrial function and antioxidant defenses, while ASA and rapamycin restore neuronal ion pump activity (e.g., Na+/K+-ATPase), mitigating oxidative stress.

**Figure 6:**
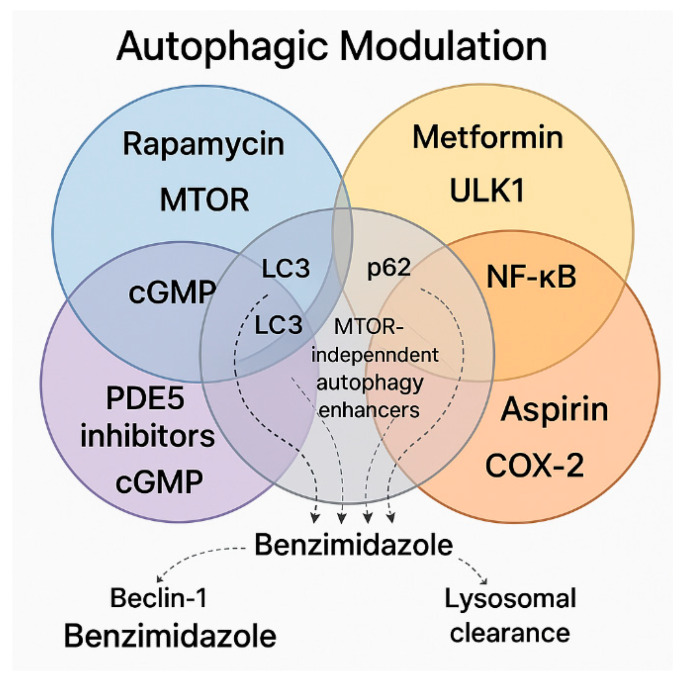
This Venn diagram illustrates the interplay of major pharmacological agents and pathways involved in autophagic modulation, highlighting both shared and unique mechanisms. Rapamycin, positioned in the blue circle, activates autophagy through the inhibition of the mTOR pathway, a well-established negative regulator of autophagy. Metformin, within the yellow circle, promotes autophagy via AMPK activation, engaging ULK1 and initiating autophagosome formation independently of mTOR. The red circle contains ASA, which modulates autophagy and inflammation by inhibiting COX-2 and NF-κB signaling. PDE5 inhibitors appear in the purple circle, acting through the cGMP-PKG pathway to stimulate autophagy in an mTOR-independent manner. Benzimidazole compounds span both the red and purple circles, implicating them in COX-2/NF-κB and cGMP-mediated mechanisms, while also being associated with enhanced lysosomal degradation. Key autophagic markers (LC3 and p62) occupy central positions, reflecting their common regulation across these pathways. Beclin-1 and lysosomal clearance are indirectly linked to benzimidazoles via dotted lines, emphasizing their contribution to mTOR-independent autophagy. The convergence of diverse signaling networks on autophagic modulation can be seen, implicating multi-target agents in therapeutic strategies. Abbreviations: mTOR—mammalian target of rapamycin; ULK1—unc-51-like autophagy-activating kinases; PDE5—phosphodiesterase-5; COX-2—cyclooxygenase-2; LC3—microtubule-associated proteins light chain 3; NF-κB—nuclear factor kappa B; cGMP—cyclic guanosine monophosphate.

These overlapping pathways (shown in Figure 6 below) highlight a multi-modal strategy where AMPK activation, mTOR inhibition, and lysosomal enhancement converge to optimize autophagy while simultaneously addressing inflammation and senescence, the key drivers of neurodegenerative and age-related diseases.

### 4.1. Key Pathways and Unique Synergies

PDE5 inhibitors, combined with mTOR inhibition by rapamycin, provide complementary mechanisms for the induction of autophagy. Additionally, Beclin-1 upregulation across multiple therapeutic agents further enhances autophagic flux, although through distinct upstream pathways. Concurrently, the suppression of the NF-κB/IL-6/TNF-α axis by rapamycin, ASA, and benzimidazoles amplifies both anti-inflammatory and anti-senescence effects, contributing to a neuroprotective environment. Notably, benzimidazole derivatives uniquely modulate autophagy through mTOR-independent mechanisms, distinguishing them from agents that rely on mTOR inhibition or AMPK activation. Instead of initiating autophagy via classical pathways involving ULK1 or Beclin-1, benzimidazoles promote autophagy by enhancing lysosomal clustering through the JIP4–TRPML1 axis. This alternative mechanism also enables them to effectively reduce cellular senescence and modulate the SASP with lower toxicity or side effects.

### 4.2. Therapeutic–Protective Composite

A combination of metformin, benzimidazole derivatives, and ASA offers a multi-targeted therapeutic strategy that could be optimized as a protective strategy to prevent or slow down the development of AD. Metformin primarily activates AMPK, a central energy sensor, leading to the inhibition of mTORC1, the activation of autophagy initiation complexes (ULK1/ATG13), and the stimulation of CMA via the TAK1–IKKα/β axis. This dual action should promote the clearance of misfolded proteins such as Aβ and hyperphosphorylated tau while simultaneously enhancing mitochondrial function through Nrf2 activation. Benzimidazole derivatives complement metformin’s upstream effects by acting downstream at the lysosomal level, where they enhance autophagosome–lysosome fusion via JIP4–TRPML1 signaling. This ensures the efficient degradation of autophagic cargo, preventing autophagic stress and facilitating the complete clearance of toxic aggregates, while additionally exerting anti-senescent and anti-inflammatory effects through NF-κB suppression and the promotion of M2 microglial polarization.

**Figure 7:**
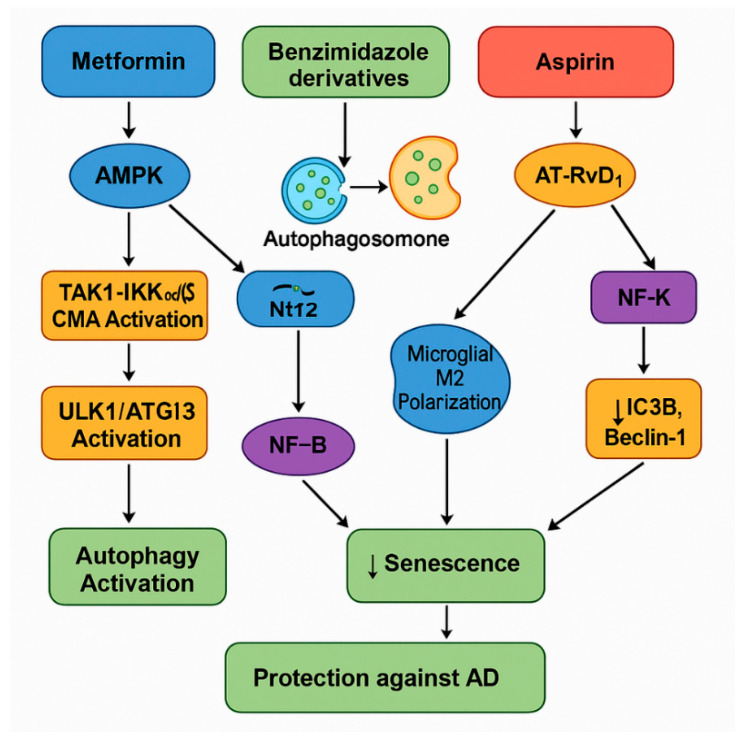
This diagram illustrates the synergistic signaling pathways activated by the combination of metformin, benzimidazole derivatives, and ASA, leading to enhanced autophagy, reduced cellular senescence, and protection against AD. Metformin activates AMPK which inhibits mTORC1, leading to the activation of the ULK1/ATG13 complex and the initiation of macroautophagy. Simultaneously, metformin triggers the TAK1–IKKα/β pathway, promoting CMA for the targeted degradation of pathogenic proteins, while also activating Nrf2-mediated antioxidant responses to protect mitochondrial function. Benzimidazole derivatives, such as albendazole and FLBZ, enhance lysosomal clustering via the JIP4–TRPML1 pathway, improving autophagosome–lysosome fusion and promoting the clearance of toxic aggregates, while independently suppressing NF-κB activity and reducing SASP factors. ASA exerts its effects by stimulating specialized pro-resolving mediators (particularly AT-RvD1), upregulating LC3B and Beclin-1 to boost autophagic flux, and concurrently inhibiting NF-κB signaling to reduce inflammation and SASP expression. Both benzimidazoles and ASA induce microglial polarization toward the anti-inflammatory M2 phenotype, creating a supportive neuroprotective environment. Together, these pathways converge to decrease cellular senescence and neuroinflammation, restore proteostasis, and promote cognitive resilience, ultimately offering protection against the progression of AD.

ASA further reinforces this therapeutic synergy by simultaneously modulating autophagy and inflammation. It stimulates the production of specialized pro-resolving lipid mediators, specifically AT-RvD1, enhancing Beclin-1 and LC3B expression to support autophagy, while independently inhibiting the COX-2 and NF-κB pathways to reduce the SASP and chronic neuroinflammation. Together, these three agents cover critical vulnerabilities in AD pathology: the initiation and completion of autophagic flux, the suppression of chronic inflammatory signaling, the reduction in cellular senescence, and the preservation of mitochondrial and synaptic function. By targeting multiple, complementary nodes in the autophagy–senescence–inflammation network, this rational drug combination holds promise for restoring cellular proteostasis and preventing the progression of neurodegeneration more effectively than monotherapies (Figure 7).

### 4.3. Further Hypothesized Combinational Strategies That Could Support AD Protection

While the combination of metformin, benzimidazole derivatives, and ASA offers a rational and mechanistically diverse approach to modulating autophagy and cellular senescence, alternative combinations may provide comparable or even superior outcomes, particularly in specific clinical contexts. One such strategy involves pairing metformin with a PDE5 inhibitor and ASA. PDE5 inhibitors, including mirodenafil and sildenafil, have demonstrated the ability to cross the blood–brain barrier and activate key signaling pathways such as cGMP–PKG–CREB and Wnt/β-catenin. These pathways not only enhance synaptic plasticity and neurovascular function but also support autophagy through mTOR-independent mechanisms. This combination would preserve AMPK activation and anti-inflammatory effects while adding potent neurovascular support, making it an attractive option for addressing vascular contributions to cognitive decline.

Alternatively, combining benzimidazole derivatives with PDE5 inhibitors and ASA may offer robust autophagy induction while minimizing potential metabolic stress linked to AMPK activation. Benzimidazoles uniquely enhance autophagosome–lysosome fusion via the JIP4–TRPML1 axis, while PDE5 inhibitors improve mitochondrial function and support cognitive resilience. Rapamycin-based combinations, such as rapamycin with benzimidazole and ASA, may further optimize autophagy by coupling upstream mTOR inhibition with enhanced lysosomal function and SASP suppression. However, given the dose-sensitive and context-dependent effects of rapamycin, its use may be most appropriate in early-stage disease or in patients with preserved lysosomal capacity. Lastly, incorporating glutaminyl cyclase inhibitors like PQ912 with metformin and ASA introduces the added benefit of inhibiting pyroglutamate-Aβ formation, potentially blocking toxic Aβ seeding. Collectively, these alternative combinations should further support personalized, multi-targeted strategies that consider disease stage, metabolic status, and drug penetrance when aiming to slow or prevent neurodegeneration in AD.

### 4.4. A Targeted and Personalized/Stratified Approach

Individuals who would most likely benefit from a combination of ASA, benzimidazole derivatives, and metformin are at risk of or in the early stages of neurodegenerative diseases, particularly AD and possibly vascular cognitive impairment. This combination targets multiple, interrelated pathological processes, including impaired autophagy, chronic inflammation, oxidative stress, and cellular senescence, which contribute to early neuronal dysfunction before irreversible damage sets in. Patients with early cognitive decline, mild cognitive impairment, or preclinical AD (amyloid-positive but cognitively intact individuals) would therefore be ideal candidates for such an intervention, as enhancing autophagic flux and reducing senescent cell burden could slow or prevent disease progression. Statistically, Parnetti et al. (2019) performed a comprehensive systematic review and meta-analysis encompassing 55 studies and found that approximately 22% of cognitively normal individuals exhibited biomarker evidence of preclinical AD. This prevalence increases with age, ranging from 16.5% at age 53 to 53% at age 86, with an estimated increase of 1.0% per year. Hence, the utilization of these protective measures will become more and more relevant over the coming decade [86]. Regarding potential plasma biomarkers, several candidates already exist that could be integrated into common clinical practice for routine testing. Elevated plasma phosphorylated tau181 (p-tau181) levels predicted accelerated cognitive decline and worsening functional outcomes across the ‘University of Pennsylvania and ADNI’ cohorts [87]. Similarly, data from the BioFINDER study showed that plasma p-tau181 could accurately predict the development of AD within six years among cognitively unimpaired individuals, with predictive accuracy that was further improved when combined with p-tau217 and the APOE genotype [88]. Additionally, a longitudinal study in two independent Chinese cohorts demonstrated that the plasma levels of Aβ42, p-tau181, and neurofilament light chain (NfL) were significantly altered up to eight years before clinical onset, supporting their utility for identifying preclinical AD [89].

Beyond AD, this combination could also be beneficial for older adults with metabolic dysfunctions (e.g., insulin resistance, type 2 diabetes, and metabolic syndrome) who are at an increased risk for neurodegeneration. Metformin’s effects on metabolism and mitochondria, combined with ASA’s anti-inflammatory action and benzimidazoles’ unique autophagy and anti-senescence effects, could help break the link between systemic metabolic disease and brain aging. Furthermore, individuals suffering from chronic low-grade neuroinflammation, post-stroke cognitive decline, or even psychiatric conditions associated with accelerated brain aging (such as schizophrenia or bipolar disorder, with evidence of mitochondrial/autophagic dysfunction) might benefit from such a broad-spectrum, multi-targeted approach.

Patient stratification for such a combinatorial therapeutic approach could be enhanced by the identification of specific molecular biomarkers associated with impaired autophagy, inflammation, and senescence. The markers of lysosomal dysfunction, such as reduced LAMP1 or cathepsin D activity, could indicate impaired autophagic flux and identify patients who would particularly benefit from the benzimidazole-mediated enhancement of autophagosome–lysosome fusion [90]. Elevated circulating or cerebrospinal fluid levels of SASP factors, including IL-6, TNF-α, and matrix metalloproteinases (MMPs), should reflect ongoing cellular senescence and chronic neuroinflammation, suggesting suitability for interventions targeting NF-κB pathways, such as ASA and benzimidazoles [8]. Furthermore, AMPK activation status, mitochondrial oxidative stress biomarkers (e.g., 8-hydroxy-2′-deoxyguanosine (8-OHdG) and mitochondrial DNA damage), and insulin resistance indices (e.g., Homeostasis Model Assessment of Insulin Resistance (HOMA-IR) scores) could guide the selection of individuals who would derive particular benefit from metformin’s metabolic and mitochondrial protective effects [34,91]. Ultimately, a precision medicine approach integrating biomarker profiling (see Table 2 below) with cognitive and imaging assessments could help optimize patient selection, maximize therapeutic efficacy, and minimize potential adverse effects when deploying this multi-targeted neuroprotective strategy.

### 4.5. Limitations and Considerations for This Approach

While the combination of ASA, benzimidazole derivatives, and metformin offers promising neuroprotective potential, careful attention to timing and dosing is critical to minimize adverse effects. As observed with rapamycin, excessive autophagy induction in the context of impaired lysosomal function can worsen pathology [29]; therefore, ensuring lysosomal readiness, potentially through the early or concurrent administration of benzimidazole compounds that enhance lysosomal fusion, appears to be essential. Similarly, chronic AMPK activation through high-dose or prolonged metformin use may have divergent effects across different brain cell types, promoting autophagy and mitochondrial health in neurons but potentially suppressing autophagic flux or inducing stress responses in astrocytes [39]. ASA, while generally well tolerated at low anti-inflammatory doses, carries a risk of gastrointestinal irritation, bleeding, and impaired wound healing, especially when combined with other agents that modify cellular stress responses. Blood–brain barrier (BBB) permeability also remains a critical challenge, as not all compounds, particularly larger or less lipophilic molecules, efficiently penetrate the CNS. Moreover, chronic or high-dose exposure to agents such as rapamycin or senolytics may induce off-target effects, including neuronal stress, immunosuppression, or altered synaptic plasticity, potentially compromising cognitive function. Additionally, the heterogeneity of CNS cell types, including neurons, microglia, astrocytes, and endothelial cells, means that the cellular response to these agents may be variable and context dependent, complicating the prediction of net effects. These concerns demonstrate the need for dose optimization, targeted delivery systems, and cell-specific analyses in future studies.

Consequently, an optimal therapeutic benefit will likely require precision dosing strategies, stage-specific timing, and the close monitoring of metabolic and inflammatory biomarkers to avoid the unintended exacerbation of neurodegeneration or systemic side effects.

### 4.6. Alternative Autophagy-Modulating Compounds for AD

Other experimental substances are currently underway that have, so far, demonstrated potential in modulating autophagy and rescuing the CNS from AD but only for in vitro or preclinical models. They include Trehalose, a natural disaccharide, which induces mTOR-independent chaperone-like autophagy, promoting the clearance of misfolded proteins like amyloid-beta and tau, with demonstrated neuroprotection in AD models [92]. Lithium, via inositol monophosphatase inhibition, enhances autophagy, reducing neurotoxic protein accumulation and showing promise in preclinical AD studies [93], whilst Latrepirdine, an antihistamine repurposed for neurodegeneration, activated autophagy and mitigated amyloid-beta pathology in experimental murine AD [94]. Other compounds include CA77.1, a synthetic CMA activator, which boosts LAMP2A expression to degrade pathogenic tau and improve cognition in AD animal studies (discussed within [95]), AUTEN-67, an autophagic flux enhancer, exhibits neuroprotection in Drosophila Huntington’s models, suggesting potential cross-application for AD [96], and finally, Humanin, a mitochondrial peptide, which stimulates CMA, counters oxidative stress, and reduces amyloid-beta, positioning it as a multi-target candidate [97]. All these molecules are currently at the very early stages of characterization.

## 5. Conclusions

The combination of metformin, benzimidazole derivatives, and ASA presents a promising multi-targeted therapeutic strategy to mitigate brain aging and prevent or delay the onset of neurodegenerative diseases such as AD. By synergistically activating autophagy, suppressing chronic inflammation, modulating cellular senescence, and supporting mitochondrial and lysosomal function, this combinatorial approach addresses a critical group of interlinked pathological processes underpinning neurodegeneration. Future research should focus on optimizing dosing regimens, elucidating cell-specific effects, and validating appropriate biomarkers to stratify patients for precision therapy. Furthermore, longitudinal preclinical and clinical studies are needed to assess the long-term efficacy and safety of this strategy, particularly considering the context-specific effects of AMPK activation and lysosomal readiness. Ultimately, integrating this combinatorial pharmacotherapy into personalized medicine frameworks could offer a transformative step toward preserving cognitive function and promoting healthy brain aging.

## 6. Future Directions

Considering the strong link between impaired autophagy, chronic inflammation, mitochondrial dysfunction, and cellular senescence in brain aging and AD, future research should aim to identify precision combination therapies that target these interconnected pathways simultaneously. The synergistic potential of metformin, benzimidazole derivatives, and ASA, as discussed, presents a framework for multi-targeted intervention. However, translating this strategy into clinical practice requires further rigorous preclinical validation using age- and pathology-specific in vivo models, and eventually, randomized clinical trials. Particular attention should be paid to timing, dosing, and patient stratification, as evidence indicates that cellular context (e.g., neuronal vs. astrocytic responses), disease stage, and lysosomal competence significantly influence therapeutic outcomes. The integration of biomarker-guided precision medicine, utilizing the markers of autophagy flux, SASP activation, oxidative stress, and metabolic dysfunction, will be essential to identify the patients most likely to benefit. Furthermore, emerging agents such as PDE5 inhibitors and glutaminyl cyclase inhibitors warrant exploration within these combination regimens for their unique neuroprotective mechanisms. Ultimately, future studies must aim to optimize synergy, minimize adverse effects, and establish long-term safety and efficacy to design novel interventions that delay or prevent cognitive decline and promote healthy brain aging.

## Figures and Tables

**Table 1:** A summary of the pharmacological characteristics, class, mode of action, and targets of the compounds described above. Abbreviations: mTOR—Mechanistic Target of Rapamycin, mTORC1—Mechanistic Target of Rapamycin Complex 1, ULK1—unc-51-like kinase 1, ATG13—autophagy-related protein 13, MEK—mitogen-activated protein kinase kinase, ERK—Extracellular Signal-Regulated Kinase, AMPK—AMP-activated protein kinase, CMA—chaperone-mediated autophagy, TAK—transforming growth factor-beta activated kinase 1, IKK—kappaB kinase, SIRT1—Sirtuin 1, Nrf2—nuclear factor erythroid 2-related factor 2, Bcl-2—B-cell lymphoma 2, PDE5—phosphodiesterase type 5, NO—nitric oxide, cGMP—cyclic guanosine monophosphate, eNOS—endothelial Nitric Oxide Synthase, CREB—cAMP Response Element-Binding Protein, BDNF—brain-derived neurotrophic factor, LC3—microtubule-associated protein 1A/1B-light chain 3, ATG5—autophagy protein 5, PGC-1α—Peroxisome Proliferator-Activated Receptor Gamma Coactivator 1-alpha, ROS—reactive oxygen species, BBB—blood–brain barrier, PKG—protein kinase G, GSK-3β—Glycogen Synthase Kinase-3 beta, FLBZ—fenbendazole, JIP4—JNK-interacting protein 4, TRPML1—transient receptor potential mucolipin 1, NLRP3—NLR family pyrin domain containing 3, NSAID—nonsteroidal anti-inflammatory drugs, COX-1/2—cyclooxygenase1/2, ASA—Acetylsalicylic Acid, AT-RvD1—aspirin-triggered resolvin D1, NLRP3—NLR family pyrin domain containing 3, TFEB—Transcription Factor EB, and PPARα—peroxisome proliferator-activated receptor alpha.

Compound	Pharmacological Class	Mechanisms of Action	Cellular Targets/Effects
Rapamycin	mTOR inhibitor (macrolide antibiotic)	Inhibits mTORC1 → activates ULK1/ATG13 → promotes autophagy; upregulates Beclin-1, reduces p62, and modulates MEK/ERK pathway	Enhances autophagy in neurons; reduces Aβ and tau; improves synaptic plasticity; modulates microglial polarization
Metformin	AMPK activator (antidiabetic biguanide)	Activates AMPK → inhibits mTORC1; induces CMA via TAK1–IKK pathway; stimulates SIRT1 and Nrf2 for antioxidant effects	Enhances autophagy (neurons and microglia); improves mitochondrial function; effects are cell-type and age dependent
Navitoclax	Senolytic (Bcl-2/Bcl-xL inhibitor)	Induces apoptosis in senescent cells by inhibiting anti-apoptotic Bcl-2 family proteins	Eliminates senescent astrocytes and endothelial cells; restores neurovascular function and cognitive performance
Sildenafil	PDE5 inhibitor	↑ NO/cGMP → activates AMPK and eNOS → inhibits mTOR; activates CREB/BDNF; modulates NF-κB; increases autophagy markers (LC3, Beclin-1, ATG5)	Promotes autophagy in neurons and glia; improves blood flow, synaptic plasticity, and cognitive performance
Tadalafil	PDE5 inhibitor	↑ NO → activates SIRT1–PGC-1α axis → improves mitochondrial function and reduces ROS	Improves mitochondrial biogenesis and function in diabetic models; potential neuroprotective effects
Mirodenafil	BBB-penetrant PDE5 inhibitor	Activates AMPK and autophagy; modulates cGMP/PKG/CREB, Wnt/β-catenin, GSK-3β, and glucocorticoid signaling	Enhances autophagy; reduces Aβ and tau; improves cognitive function and synaptic signaling
FLBZ/Albendazole	Benzimidazole derivatives	Promote lysosomal clustering, JIP4–TRPML1 activation, enhance autophagosome–lysosome fusion; modulate NF-κB and NLRP3 pathways	Induce autophagy without mTOR inhibition; reduce SASP and neuroinflammation; clear protein aggregates
Acetylsalicylic acid (ASA)	NSAID/COX-1/2 inhibitor	Induces autophagy via COX-2 inhibition and AT-RvD1 generation; inhibits NF-κB and NLRP3; activates TFEB via PPARα	Promotes clearance of Aβ/tau; reduces senescence and inflammation; preserves synaptic function

**Table 2:** Potential biomarkers for patient stratification and therapeutic targeting with metformin, benzimidazole derivatives, and ASA. Abbreviations: LAMP1—Lysosomal-Associated Membrane Protein 1, TRPML1—transient receptor potential mucolipin 1, HOMA-IR—Homeostasis Model Assessment of Insulin Resistance, 8-OHdG—8-hydroxy-2′-deoxyguanosine, DNA—Deoxyribonucleic Acid, Nrf2—nuclear factor erythroid 2-related factor 2, mTORC1—Mechanistic Target of Rapamycin Complex 1, AMPK—AMP-activated protein kinase, NF-κB—nuclear factor kappa B, IL-6—Interleukin-6, TNF-α—Tumor Necrosis Factor alpha, MMP—matrix metalloproteinase, ASA—acetylsalicylic acid, and SASP—senescence-associated secretory phenotype.

Biomarker	Pathway Dysfunction Indicated	Relevant Drug	Therapeutic Action
↓ LAMP1, ↓ Cathepsin D	Impaired autophagosome–lysosome fusion	Benzimidazole derivatives	Enhance autophagic degradation via JIP4–TRPML1 pathway
↑ IL-6, ↑ TNF-α, ↑ MMPs	Chronic inflammation and SASP activation	ASA and benzimidazoles	Suppress NF-κB signaling and SASP factors
↑ NF-κB activation (p65 subunit)	Inflammatory and senescence pathways	ASA and benzimidazoles	NF-κB inhibition and anti-inflammatory action
↓ AMPK activation, ↑ mTORC1 activity	Impaired autophagy initiation and metabolic stress	Metformin	AMPK activation and mTORC1 inhibition to restore autophagy
↑ 8-OHdG, mitochondrial DNA damage	Oxidative stress and mitochondrial dysfunction	Metformin	Nrf2-mediated antioxidant defense and mitochondrial support
↑ HOMA-IR score, insulin resistance	Metabolic dysfunction	Metformin	Improvement of insulin sensitivity and energy homeostasis

## Data Availability

No new data were created or analyzed in this study.

## Abbreviations

The following abbreviations are used in this manuscript:

8-OHdG	8-hydroxy-2′-deoxyguanosine
AChE	Acetylcholinesterase
AD	Alzheimer’s disease
Akt	Protein Kinase B
ALS	Amyotrophic Lateral Sclerosis
AMPK	AMP-activated protein kinase
APOE4	Apolipoprotein E4
APP	Amyloid Precursor Protein
ASA	Acetylsalicylic Acid
AT-RvD1	Aspirin-Triggered Resolvin D1
Aβ	β-amyloid
BBB	Blood–Brain Barrier
BChE	Butyrylcholinesterase
BDNF	Brain-Derived Neurotrophic Factor
BPH	Benign Prostatic Hyperplasia
cGMP	Cyclic Guanosine Monophosphate
CMA	Chaperone-Mediated Autophagy
COX	Cyclooxygenase
CREB	cAMP Response Element-Binding Protein
ED	Erectile Dysfunction
FLBZ	Flubendazole
GGPP	Geranylgeranyl Pyrophosphate
GSK3β	Glycogen Synthase Kinase 3 Beta
HMGB1	High mobility group box 1 protein
HOMA-IR	Homeostasis Model Assessment of Insulin Resistance
IL-6	Interleukin-6
LC3	Microtubule-associated protein 1 light chain 3
LTP	Long-Term Potentiation
MAPK	Mitogen-Activated Protein Kinase
MMP	Matrix Metalloproteinase
mTOR	Mammalian Target of Rapamycin
mTORC1	Mammalian Target of Rapamycin Complex 1
NF-κB	Nuclear factor kappa B
NO	Nitric Oxide
Nrf2	Nuclear factor erythroid 2-related factor 2
OXPHOS	Oxidative Phosphorylation
PAH	Pulmonary Arterial Hypertension
PD	Parkinson’s disease
PDE5	Phosphodiesterase-5
PGC-1α	Peroxisome Proliferator-Activated Receptor Gamma Coactivator 1-alpha
PPAR-γ	Peroxisome proliferator-activated receptor gamma
ROS	Reactive Oxygen Species
RPE	Retinal Pigment Epithelial Cells
SASP	Senescence-Associated Secretory Phenotype
SIRT1	Sirtuin 1
SPMs	Specialized Pro-resolving Mediators
TNF-α	Tumor Necrosis Factor alpha
ULK-1	Unc-51-Like Autophagy Activating Kinase 1
